# Clinical evaluation of laryngeal mask airways in video-assisted thoracic surgery: a meta-analysis of randomized controlled trials

**DOI:** 10.1186/s13019-024-02840-6

**Published:** 2024-06-24

**Authors:** Kai Luo, Kaiming Chen, Yu Li, Yang Ji

**Affiliations:** 1 Department of Anesthesiology, West China Hospital of Stomatology, Sichuan University, Chengdu, China; 2 Department of Anesthesiology, West China Hospital, Sichuan University, Chengdu, China

**Keywords:** Endotracheal intubation, Laryngeal mask airway, Video-assisted thoracic surgery

## Abstract

**Background:**

Endotracheal intubation is often associated with postoperative complications such as sore throat discomfort and hoarseness, reducing patient satisfaction and prolonging hospital stays. Laryngeal mask airway (LMA) plays a critical role in reducing airway complications related to endotracheal intubation. This meta-analysis was performed to determine the efficacy and safety of LMA in video-assisted thoracic surgery (VATS).

**Methods:**

The PubMed, Embase, Cochrane Library, Medline and Web of Science databases were searched for eligible studies from inception until October 5, 2023. Cochrane’s tool (RoB 2) was used to evaluate the possibility biases of RCTs. We performed sensitivity analysis and subgroup analysis to assess the robustness of the results.

**Results:**

Seven articles were included in this meta-analysis. Compared with endotracheal intubation, there was no significant difference in the postoperative hospital stay (SMD = -0.47, 95% CI = -0.98–0.03, *P* = 0.06), intraoperative minimum SpO2 (SMD = 0.00, 95% CI = -0.49–0.49, *P* = 1.00), hypoxemia (RR = 1.00, 95% CI = 0.26–3.89, *P* = 1.00), intraoperative highest PetCO2 (SMD = 0.51, 95% CI = -0.12–1.15, *P* = 0.11), surgical field satisfaction (RR = 1.01, 95% CI = 0.98–1.03, *P* = 0.61), anesthesia time (SMD = -0.10, 95% CI = -0.30–0.10, *P* = 0.31), operation time (SMD = 0.06, 95% CI = -0.13–0.24, *P* = 0.55) and blood loss (SMD =- 0.13, 95% CI = -0.33–0.07, *P* = 0.21) in LMA group. However, LMA was associated with a lower incidence of throat discomfort (RR = 0.28, 95% CI = 0.17–0.48, *P* < 0.00001) and postoperative hoarseness (RR = 0.36, 95% CI = 0.16–0.81, *P* = 0.01), endotracheal intubation was found in connection with a longer postoperative awake time (SMD = -2.19, 95% CI = -3.49 – -0.89, *P* = 0.001).

**Conclusion:**

Compared with endotracheal intubation, LMA can effectively reduce the incidence of throat discomfort and hoarseness post-VATS, and can accelerate the recovery from anesthesia. LMA appears to be an alternative to endotracheal intubation for some specific thoracic surgical procedures, and the efficacy and safety of LMA in VATS need to be further explored in the future.

**Supplementary Information:**

The online version contains supplementary material available at 10.1186/s13019-024-02840-6.

## Introduction

Video-assisted thoracic surgery (VATS) alleviates the major surgical trauma of traditional thoracotomy and opens a new era of minimally invasive thoracic surgery [1]. With the optimization of direct visual panoramic view of the half chest in VATS and the advancement of fine endoscopic techniques, VATS have gradually been introduced into complex thoracic surgery, which includes removing mediastinal mass, performing pneumonectomy and excising chest wall disease [2, 3]. Compared with traditional thoracotomy, VATS is the preferred surgical method for thoracic surgery, with minimal trauma and incision, and reliable postoperative pain reduction and healing [4]. Anatomically, bilateral lungs are connected by bronchus, and a variety of lung isolation techniques need to be considered when performing lung tissue resection, whether in traditional thoracotomy or VATS, to protect healthy lungs from invasion of the affected side of the lesion, while providing a clear and operable surgical area.

Double-lumen tubes (DLTs) and endotracheal tubes (ETTs) combined with endobronchial blockers can achieve reliable and effective lung isolation, which has become the mainstream anesthesia methods for thoracic surgery. However, these two techniques can induce a variety of intubation-related complications, including airway injury, postoperative sore throat, hoarseness, stress-related lung injury, lung infection, and bronchospasm [5–8]. To address these issues, several surgeons and anesthesiologists have attempted to introduce nontracheal intubation techniques into thoracic surgery airway management. The laryngeal mask airway (LMA) has shown broad application prospects in VATS because of its advantages in reducing small respiratory tract injuries and ensuring safe and effective airway control [9–11].

LMA anesthesia is a technique for supraglottic airway control using LMA, which has the advantages of less mechanical injury, easy to perform, less postoperative complications, quick recovery and low cost [12, 13]. In addition, use of LMA is associated with reduced perioperative respiratory adverse events (laryngospasm and bronchospasm, etc.) compared with endotracheal tubes in children [14]. LMA involves optimized anesthesia and surgical management, enhanced postoperative recovery and early discharge. Although, the routine use of non-intubated anesthesia in VATS cannot be recommended, LMA still shows a promising prospect in the field of enhanced recovery after surgery (ERAS) in VATS [15–17]. However, the application of LMA in thoracic surgery anesthesia is limited owing to the requirement of one-lung ventilation technique. And prolonged ventilation also results in observed obvious mucosal damage [18]. Moreover, the combination of LMA and endobronchial blockers presents significant challenges for anesthesiologists and airway management. Therefore, in clinical practice, LMA anesthesia may not be recommended for prolonged surgery, and for those less invasive thoracic surgery procedures that are emerging and rapidly spreading around the world, LMA anesthesia may show the predominant advantage.

LMA provides a good experience for some patients who underwent various short surgeries. A survey amongst the European Society of Thoracic Surgeons (ESTS) members has been performed to investigate the currents trends, as well as potential for future expansion of non-intubated thoracic surgery performed under spontaneous ventilation. The results indicate that the preferred types of anesthesia were intercostal blocks, followed by use of LMA with sedation. And the majority of responders indicated that multiple comorbidity, poor pulmonary function and advanced age should be considered as the main eligibility criteria for non-intubated thoracic surgery [19]. For patients with difficulty in intubation for lung isolation such as upper tracheal stenosis, LMA provides a useful alternative to the endotracheal intubation. However, in thoracic surgery, continuous hypoxemia, carbon dioxide retention, difficulty in airway management and the need for experienced anesthesiologists still limit the widespread application of LMA in clinical practice. Whether LMA offers a good benefit-to-risk ratio for certain patient groups has not been determined. Therefore, it is necessary for us to conduct a systematic review and meta-analysis of previous studies to evaluate the safety and effectiveness of LMA in thoracic surgery.

## Materials and methods

The reporting of this systematic review and meta-analysis followed the latest PRISMA recommendations [20], and a protocol was registered with Prospero (registration number: CRD42023493677).

### Eligibility criteria

This systematic review and meta-analysis included clinical studies comparing the safety and efficacy of LMA and tracheal intubation in VATS. There was no language restriction, and we did not seek any unpublished trials. The final decision for inclusion was determined based on consensus among the four researchers. Our inclusion criteria were as follows:

1. Population: patients who underwent VATS.
2. Intervention: use of LMA (regardless of manufacturer), and comparison: the use of ETT or DLT.
3. The outcomes included at least one of the following: throat discomfort, hoarseness, postoperative hospital stay, postoperative awake time, intraoperative minimum pulse blood oxygen saturation (SpO2), intraoperative highest peak of end tidal CO2 pressure (PetCO2), hypoxemia, surgical field satisfaction, anesthesia time, operation time and blood loss.
4. The study design was a randomized controlled trial.

The exclusion criteria were as follows:

1. The procedure was reversed to thoracotomy for any reason.
2. The LMA or intubation was preserved, and the patients were then transferred to the intensive care unit (ICU) after thoracoscopic surgery.
3. Unpublished studies in peer-reviewed journals, reviews, cases, comments, abstracts or letters were excluded.

### Information sources and search methods

Two researchers (LK and CMK) independently searched the PubMed, Embase, Cochrane Library, Medline and Web of Science databases for eligible studies from inception until October 5, 2023. The following keywords and medical subject headings were used: (“laryngeal mask” or “laryngeal mask airway” or “LMA”) and (“thoracoscopic” or “thoracic surgery” or “thoracic surgery video-assisted” or “VATS”). We provided a PRISMA checklist and the details of the search strategy can be found in appendix 1.

### Study selection

Two researchers (LK and CMK) independently removed duplicates, selected studies eligible for full text reading by reviewing titles and abstracts, and then conducted the final literature screening according to the inclusion and exclusion criteria. Any disagreements in this study selection process were resolved by consultation with a third author (JY).

### Data collection

The data were extracted independently by two researchers (LK and CMK) and presented in the form of Microsoft Excel spreadsheets, and the study selection and data extraction processes were cross-checked. Any disagreements during this process were resolved through discussion, and if necessary, a third author (JY) was consulted. The following data were collected from each study: the first author, year, number of patients, type of VATS, postoperative hospital stay, postoperative awake time, surgical field satisfaction, anesthesia time, operation time, intraoperative minimum SpO2, blood loss and any reported complications (throat discomfort, hoarseness, intraoperative highest PetCO2, hypoxemia).

Most of the data were retrieved directly from the original text, for those who were presented as a graph, we contacted the corresponding author or used Plot digitizer. We extracted continuous data as the mean and standard deviation, if the median/quartile interval or range was displayed, we converted the data to mean and standard deviation using statistical formulas [21, 22].

### Risk of bias and quality of evidence assessment

The risk of bias and quality of evidence assessment were performed independently by two researchers (LK and CMK), similarly, any disagreements during the methodological quality evaluation cross-check process were resolved by discussion or consultation with a third author (JY). Two authors employed the Cochrane’s tool (RoB 2) [23] to evaluate the possibility of biases. Cochrane’s tool (RoB 2) included randomized sequence generation, allocation concealment, blinding of participants and personnel, blinding of outcome assessment, incomplete outcome data, selective reporting, and other potential biases. Each was graded ‘high’, ‘low’ or ‘unclear’, which reflected a high, low and uncertain risk of bias, respectively.

The quality and strength of the evidence for the outcome were based on the guidelines of the Grades of Recommendation, Assessment, Development and Evaluation (GRADE) [24]. We assessed the risk of bias, inconsistency, indirectness, imprecision and other considerations, and the results were rated as very low, low, moderate or high.

### Statistical analysis

We used RevMan 5.4 for this meta-analysis. The mean difference (MD) with a corresponding 95% confidence interval (CI) was used to express continuous variables, and the standardized mean difference (SMD) with a corresponding 95% CI was used to express nonnormally distributed continuous data. We converted the median, range and interquartile range to the mean and standard deviation using the statistical formula [21, 22] for continuous data not in the form of the mean or standard deviation. For binary variables, the relative risk (RR) value with the corresponding 95% CI was calculated. The I ^2^ statistic was used to assess heterogeneity across the included studies with significance predefined at I ^2^ > 50%, and *P* < 0.05 was considered to indicate statistical significance. Since I ^2^ was greater than 50%, it was considered as heterogeneous, the random-effects model was used, and a fixed effects model was used if the I ^2^ value was less than 50%. We performed a sensitivity analysis for outcomes by using the leave-one-out approach to identify the possible sources of between-study heterogeneity and to assess robustness. However, it was not possible to perform a sensitivity analysis when comparing three or fewer studies.

### Subgroup analysis

We performed a subgroup analysis according to the type of tubes (ETT or DLT), the type of ventilation applied (mechanical ventilation or spontaneous ventilation) and the use of LMA associated with bronchial blockers (BB) or not.

## Results

**Fig. 1:**
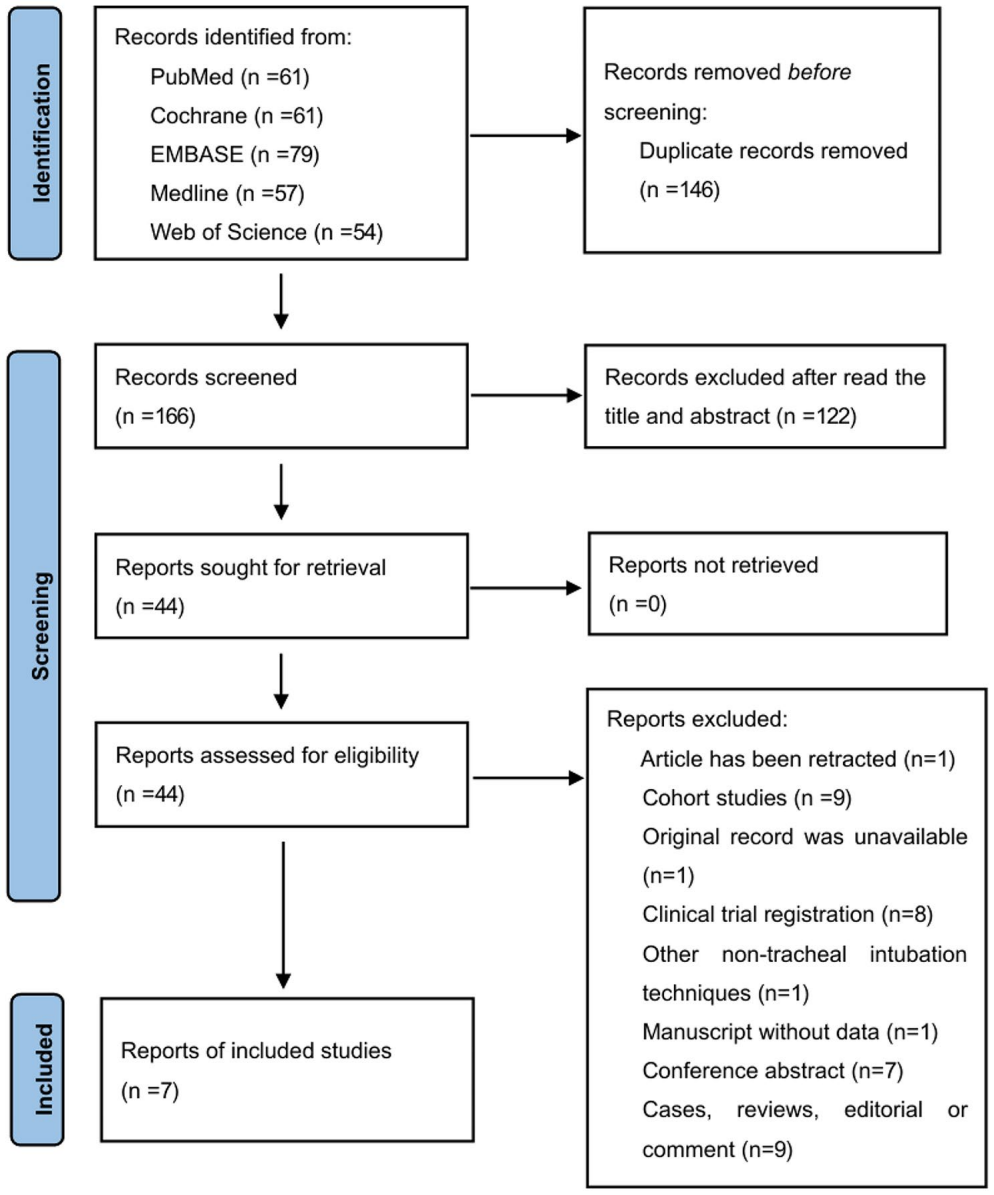
Flowchart showing selection of articles for review

We initially retrieved a total of 312 potentially eligible records from the PubMed (*n* = 61), Cochrane Library (*n* = 86), EMBASE (*n* = 79), Medline (*n* = 57), and Web of Science (*n* = 54) databases. We deleted duplicate records from different databases (*n* = 146), with 44 remaining after reviewing titles and abstracts. Finally, a total of seven RCTs were included after detailed assessment of the full text [13, 25–30]. A flow diagram summarizing the process of study selection is demonstrated in Fig. 1.

The characteristics of the included studies are shown in Table 1. The total sample size of participants ranged from 55 to 217. These studies were performed in different countries, six of the studies were from China [13, 25–27, 29, 30], and the remaining one was from Japan [28]. Two articles [13, 26] included a total of 162 patients in the LMA group without added muscle relaxants and who received continuous spontaneous ventilation, while the remaining 162 patients in the intubation group and all patients in five studies [25, 27–30] received mechanical ventilation.

**Table 1:** Study characteristics

Author, year	Country	Group	Ventilation	Sample size	Age(years)	Sex, male, %	Type of surgery
Kaican, 2013	China	LMA	Mechanical	30	23.5 ± 10.6	19 (63.3%)	VATS for wedge resection
		DLT	Mechanical	30	22.1 ± 9.7	13 (43.3%)	
Chengya, 2022	China	LMA	spontaneous	109	49.0 (39.0–58.0) ^a^	15 (16.0%)	VATS for wedge resection, segmentectomy, or lobectomy
		DLT	Mechanical	108	50.0 (42.0–60.0) ^a^	20 (19.0%)	
Qiong, 2014	China	LMA + BB	Mechanical	26	55.0 ± 15.0	18 (69.2%)	VATS for day-case thoracic surgeries
		DLT	Mechanical	29	57.0 ± 13.0	17 (58.6%)	
Nakanishi, 2023	Japan	LMA + BB	Mechanical	49	67.0 ± 11.0	26 (53.1%)	VATS for lobectomy, segmentectomy and partial resection
		DLT	Mechanical	49	64.0 ± 13.0	25 (51.0%)	
Songsong, 2018	China	LMA	Mechanical	30	21.0 ± 3.2	19 (63.3%)	VATS for Nuss repair
		ETT	Mechanical	30	20.0 ± 3.8	17 (56.7%)	
Shaolin, 2013	China	LMA + BB	Mechanical	50	43.2 ± 14.7	35 (70.0%)	VATS for pulmonary bulla, lobectomy, biopsy, and mediastinal mass excision
		ETT + BB	Mechanical	50	45.4 ± 11.8	36 (72.0%)	
Kaikai, 2022	China	LMA	spontaneous	53	51.0 ± 19.3	33 (62.3%)	VATS for wedge resection
		ETT	Mechanical	54	50.5 ± 16.3	30 (55.6%)	

LMA: laryngeal mask airway; DLT: double-lumen tube; ETT: endotracheal intubation; VATS: video-assisted thoracoscopic surgery; BB: bronchial blocker

^a^ presented as the median (interquartile range)

### Risk of bias and quality of evidence assessment

**Fig. 2:**
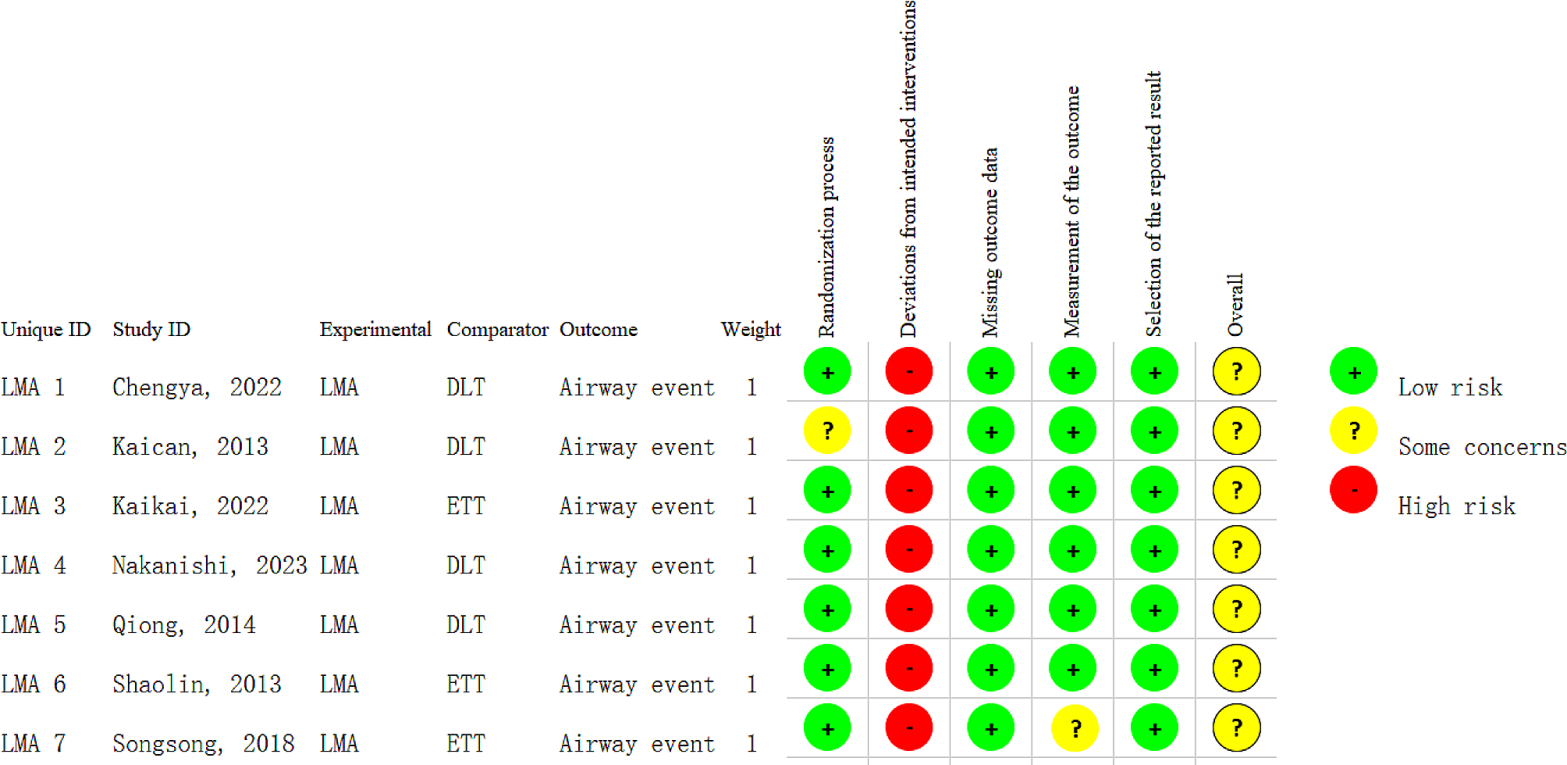
The risk of bias assessment for the included studies

The risk of bias assessment for the included studies is shown in Fig. 2, in which the methodological component quality was considered to be low or moderate. We determined that assessors of postoperative outcomes in all the studies were blinded. However, for obvious technical reasons, the operators who performed the airway maneuvers were unblinded in any of the studies. In one study, the bias arising from the other bias was judged to be high, as all the clinical data were collected from the institutional database and medical records that lacked detailed descriptions.

Based on the GRADE approach, we found the quality of evidence for postoperative hospital stay, postoperative awake time and intraoperative minimum SpO2 was low, due to high risk of bias, high heterogeneity, or small sample size. The quality of evidence was moderate for hoarseness and intraoperative highest PetCO2, and the quality of evidence for throat discomfort, hypoxemia, surgical field satisfaction, anesthesia time, operation time and blood loss was high (appendix 2).

### Postoperative outcomes

#### Postoperative hospital stay

**Fig. 3:**
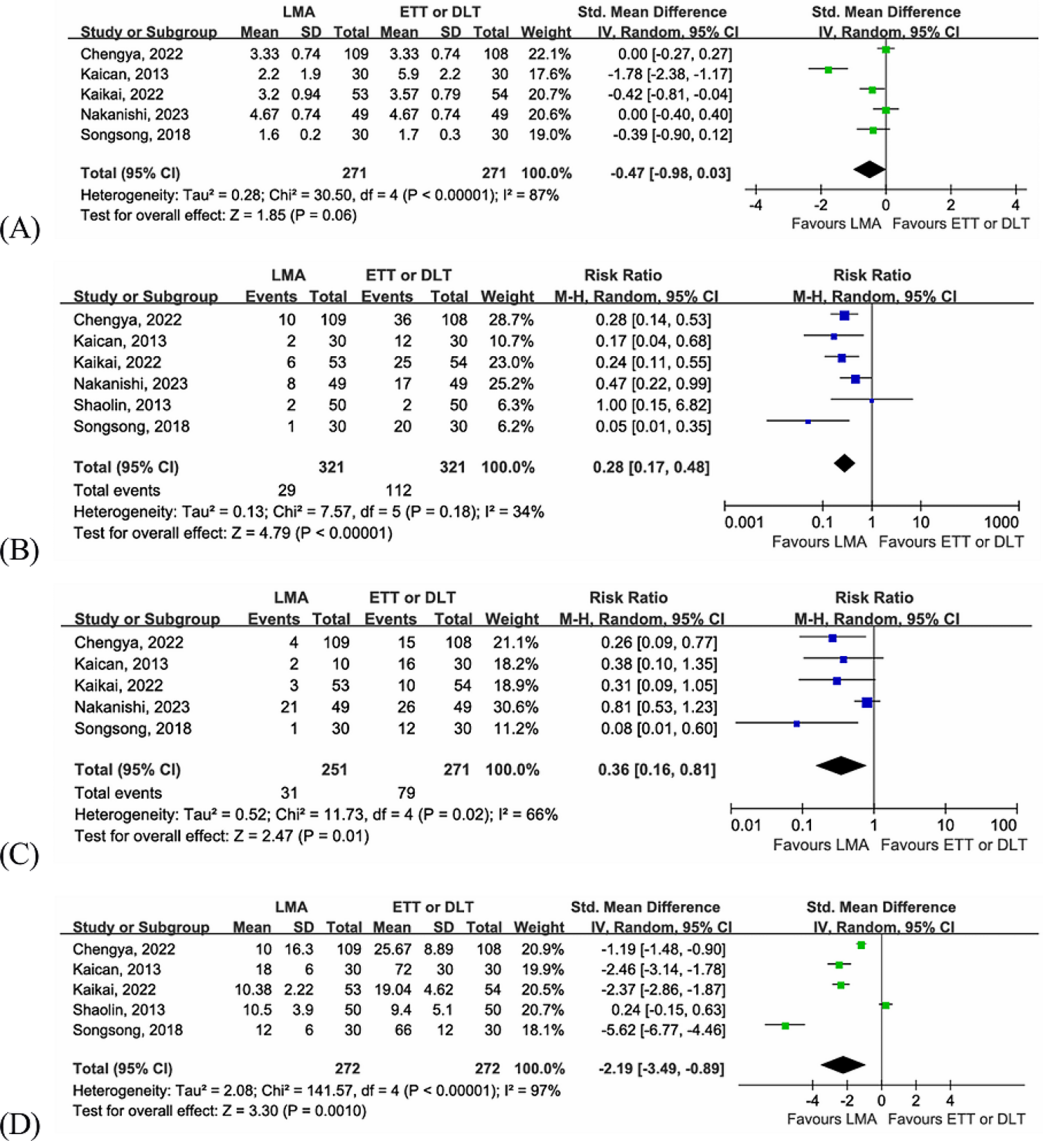
Forest plots for postoperative outcomes. (**A**) postoperative hospital stay, (**B**) throat discomfort, (**C**) hoarseness, (**D**) postoperative awake time

Five studies [13, 25, 26, 28, 29] included a total of 542 patients reported the difference in postoperative hospital stay between the LMA group and the intubation group. Meta-analysis indicated that there were no significant differences between the two groups (SMD = -0.47, 95% CI = -0.98–0.03, *P* = 0.06). There was significant heterogeneity among the included articles (I ^2^ = 87%, *P* < 0.00001, Fig. 3A). We found one of the articles [25] was conducted earlier than others, although the type of surgery in this study was simple bullectomy, we did not find that the average length of hospital stay was significantly lower than in other studies. After excluding this study, there was still no significant difference in postoperative hospital stay between the two groups (SMD = -0.16, 95% CI = -0.39–0.07, *P* = 0.16), but heterogeneity was significantly reduced (I ^2^ = 34%, *P* = 0.21). It is reasonable to hypothesis that possible heterogeneity is derived from the medical conditions at that time and small sample size.

#### Throat discomfort

Six studies [13, 25, 26, 28–30] with 642 patients compared the incidence of postoperative throat discomfort. Compared with those in the DLT or ETT groups, patients in the LMA group were more likely to avoid throat discomfort (RR = 0.28, 95% CI = 0.17–0.48, *P* < 0.00001), with acceptable heterogeneity between the articles (I ^2^ = 34%, *P* = 0.18, Fig. 3B).

#### Hoarseness

Five hundred and twenty-two patients in five studies [13, 25, 26, 28, 29] compared the incidence of postoperative hoarseness. As shown in the forest plot, the incidence of postoperative hoarseness in the LMA group was lower than that in the intubation group (RR = 0.36, 95% CI = 0.16–0.81, *P* = 0.01), and significant heterogeneity was observed (I ^2^ = 66%, *P* = 0.02, Fig. 3C). Nakanishi et al. [28] reported that the incidence of postoperative hoarseness was similar between the LMA group and the intubation group, which might be related to the operation time and duration of intubation. After excluding this study, the incidence of postoperative hoarseness was lower in the LMA group (RR = 0.27, 95% CI = 0.14–0.51, *P* < 0.0001), and without heterogeneity was observed (I ^2^ = 0%, *P* = 0.63).

Postoperative awake time.

Five studies [13, 25, 26, 29, 30] with 544 patients reported postoperative awake time. Compared with those in the intubation group, patients in the LMA group reported a shorter postoperative recovery time, indicating a shorter time to remove the artificial airway after surgery (SMD = -2.19, 95% CI = -3.49 – -0.89, *P* = 0.001). There was significant heterogeneity between the two groups among the included studies (I ^2^ = 97%, *P* < 0.00001, Fig. 3D).

### Intraoperative outcomes

#### Intraoperative minimum SpO2

**Fig. 4:**
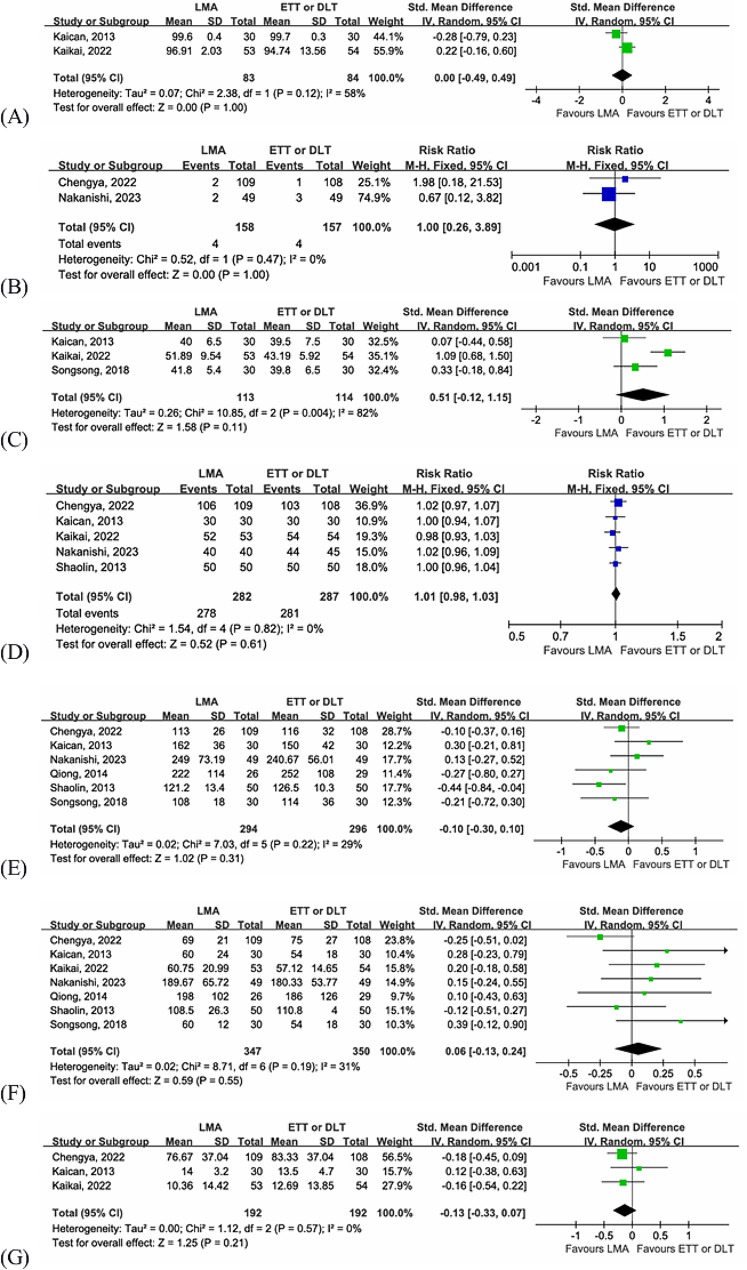
Forest plots for intraoperative outcomes. (**A**) intraoperative minimum SpO2, (**B**) hypoxemia, (**C**) intraoperative highest PetCO2, (**D**) surgical field satisfaction, (**E**) anesthesia time, (**F**) operation time, (**G**) blood loss

There were two articles [13, 25] documenting intraoperative minimum SpO2, in which data were reported for 167 patients. Pooled analysis revealed no significant difference in the intraoperative minimum SpO2 between the LMA group and the intubation group (SMD = 0.00, 95% CI = -0.49–0.49, *P* = 1.00), with high heterogeneity between the articles (I ^2^ = 58%, *P* = 0.12, Fig. 4A).

#### Hypoxemia

A total of two articles [26, 28] reported the incidence of hypoxemia, with a total of 315 patients. Pooled analysis of the data showed that there was no significant difference in the incidence of intraoperative hypoxemia between the LMA group and the intubation group (RR = 1.00, 95% CI = 0.26–3.89, *P* = 1.00), and no heterogeneity was noted (I ^2^ = 0%, *P* = 0.47, Fig. 4B).

Intraoperative highest PetCO2.

Three articles [13, 25, 29] involving a total of 227 patients reported the intraoperative highest PetCO2. In our meta-analysis, we found no significant difference in intraoperative highest PetCO2 between the LMA and intubation groups (SMD = 0.51, 95% CI = -0.12–1.15, *P* = 0.11), with significant heterogeneity between the articles (I ^2^ = 82%, *P* = 0.004, Fig. 4C). Kaikai et al. [13] included patients who underwent thoracoscopic pulmonary wedge resection. In this study, patients in the LMA group with spontaneous ventilation, the intraoperative highest PetCO2 was significantly higher than that in the intubation group with mechanical ventilation. We considered the main source of heterogeneity to be the type of ventilation.

#### Surgical field satisfaction

The data on surgical field satisfaction were extracted from five studies [13, 25, 26, 28, 30] with 569 patients reporting intraoperative surgeon satisfaction with the surgical field and lung collapse. In our meta-analysis, we found no difference in surgeon satisfaction with the surgical field and lung collapse between the LMA and intubation groups (RR = 1.01, 95% CI = 0.98–1.03, *P* = 0.61). Among all included articles, the identity was high and there was no heterogeneity (I ^2^ = 0%, *P* = 0.82, Fig. 4D).

#### Anesthesia time

There were six articles [25–30] with a total of 590 patients reporting the anesthesia time. Patients in the LMA group were found to have the same duration of anesthesia time as those in the intubation group (SMD = -0.10, 95% CI = -0.30–0.10, *P* = 0.31), with slight heterogeneity among the included articles (I ^2^ = 29%, *P* = 0.22, Fig. 4E).

#### Operation time

Seven studies [13, 25–30] with a total of 697 patients reported the operation time. Compared with those in the intubation group, patients in the LMA group were found to have the same duration of operation (SMD = 0.06, 95% CI = -0.13–0.24, *P* = 0.55). There was slight heterogeneity among the included literature (I ^2^ = 31%, *P* = 0.19, Fig. 4F).

#### Blood loss

Three studies [13, 25, 26] including 384 patients evaluated blood loss between the LMA and intubation groups, There was no significant difference was observed (SMD = -0.13, 95% CI = -0.33–0.07, *P* = 0.21), and no significant heterogeneity was observed among the included articles (I ^2^ = 0%, *P* = 0.57, Fig. 4G).

### Sensitivity analysis

We performed a sensitivity analysis to quantify the source of heterogeneity and assess the robustness of the results (appendix 3). Sensitivity analysis for outcomes with significant heterogeneity was performed by excluding each individual study, and no significant effects on the pooled results were found.

### Subgroup analysis

We were unable to perform a subgroup analysis according to the type of surgery, due to the large number of thoracic surgical procedures in the studies we included. Subgroup analysis was performed according to tracheal tube and mode of ventilation. The results of subgroup analysis were consistent with pooled analysis, with significantly reduced heterogeneity between the included studies. The details are summarised in Table 2.

**Table 2:** Meta-analysis of subgroups

Outcomes	Number of studies	Number of patients	OR/SMD	95% CI	*P*	I ^2^ (%)
mechanical ventilation						
Postoperative hospital stay	4	486	0.58	0.32, 1.06	0.08	41
Throat discomfort	2	302	1.02	0.98, 1.06	0.35	0
Hoarseness	3	304	-0.18	-0.55, 0.19	0.34	58
Postoperative awake time	5	646	-0.54	-1.26, 0.18	0.14	95
Intraoperative highest PetCO2	4	694	-0.11	-0.26, 0.04	0.14	0
Surgical field satisfaction	3	507	0.32	0.17, 0.58	0.0002	36
Anesthesia time	2	315	0.51	0.16, 1.60	0.25	76
Operation time	6	838	-0.35	-0.82, 0.13	0.15	90
**spontaneous ventilation**						
Postoperative hospital stay	2	324	-0.19	-0.60, 0.22	0.37	68
Throat discomfort	2	324	0.26	0.16, 0.44	< 0.00001	0
Hoarseness	2	324	0.28	0.13, 0.63	0.002	0
Postoperative awake time	2	324	-1.76	-2.91, -0.61	0.003	94
Surgical field satisfaction	2	324	1.01	0.97, 1.05	0.74	15
Operation time	2	324	-0.05	-0.48, -0.39	0.84	72
Blood loss	2	324	-0.17	-0.39, 0.04	0.12	0
**LMA vs. DLT**						
Postoperative hospital stay	3	375	-0.55	-1.44, 0.34	0.22	93
Throat discomfort	3	375	0.32	0.20, 0.52	< 0.00001	5
Hoarseness	3	355	0.49	0.22, 1.12	0.09	61
Postoperative awake time	2	277	-1.79	-3.03, -0.54	0.005	91
Hypoxemia	2	315	1.00	0.26, 3.89	1.00	0
Surgical field satisfaction	3	362	1.02	0.98, 1.05	0.37	0
Anesthesia time	4	430	-0.01	-0.21, 0.19	0.92	8
Operation time	4	430	0.02	-0.25, 0.28	0.90	40
Blood loss	2	277	-0.11	-0.36, 0.14	0.40	6
**LMA vs. ETT**						
Postoperative hospital stay	2	167	-0.14	-0.72, -0.10	0.009	0
Throat discomfort	3	267	0.23	0.06, 0.94	0.04	60
Hoarseness	2	167	0.20	0.06, 0.68	0.01	21
Postoperative awake time	3	267	-2.53	-5.20, 0.15	0.06	98
Intraoperative highest PetCO2	2	167	0.73	-0.02, 1.47	0.06	81
Surgical field satisfaction	2	207	0.99	0.96, 1,02	0.56	0
Anesthesia time	2	160	-0.35	-0.66, -0.04	0.03	0
Operation time	3	267	0.13	-0.15, 0.41	0.37	25
**LMA**						
Postoperative hospital stay	4	444	-0.16	-1.24, 0.03	0.06	89
Throat discomfort	4	444	0.23	0.14, 0.36	< 0.00001	3
Hoarseness	4	424	0.27	0.14, 0.51	< 0.0001	0
Postoperative awake time	4	444	-2.80	-4.11, -1.49	< 0.0001	96
Intraoperative minimum SpO2	2	167	0.00	-0.49, 0.49	1.00	58
Intraoperative highest PetCO2	3	227	0.51	-0.12, 1.15	0.11	82
Surgical field satisfaction	3	384	1.01	0.97, 1.04	0.76	0
Anesthesia time	3	337	-0.04	-0.29, 0.21	0.75	16
Operation time	4	444	0.11	-0.21, 0.43	0.51	61
**LMA + BB**						
Throat discomfort	2	198	0.52	0.26, 1.04	0.06	0
Surgical field satisfaction	2	185	1.01	0.97, 1.05	0.60	0
Anesthesia time	3	253	-0.19	-0.55, 0.17	0.31	51
Operation time	3	253	0.03	-0.21, 0.08	0.79	0

SpO2: pulse blood oxygen saturation; PetCO2: peak of end tidal CO2 pressure; OR: odds ratio; SMD: standardized mean difference; CI: confidence interval; BB: bronchial blocker

## Discussion

The incidence of airway complications post-VATS is higher than that of non-thoracic surgery. Currently, some studies are exploring the application of nonintubation techniques in VATS [31]. LMAs has been commonly used in various short procedures, however, the safety and effectiveness in VATS still need further discussion. A total of 697 patients in seven original studies were included in this systematic review and meta-analysis, which were mainly completed in China. These studies comprehensively and systematically evaluated the safety and efficacy of LMA versus DLT/ETT in VATS. The results indicated that there was no statistically significant difference in postoperative hospital stay between the LMA and intubation groups. However, the lack of a significant difference does not imply equivalence, most of the studies and overall trends indicate that the postoperative hospital stay was shorter in the LMA group than that in the endotracheal intubation group. We consider the regions of studies performed are not universally and small sample size may cause a potential misunderstanding to the outcome.

Compared with that in the intubation group, the rate of throat discomfort, hoarseness was lower in the LMA group, and LMA group with the shorter postoperative awake time. The two groups had similar intraoperative minimum SpO2, hypoxemia, intraoperative highest PetCO2, surgical field satisfaction, operation time, anesthesia time and blood loss. We found that most of the operation time was within two hours, and the type of operation was mainly minor VATS, such as wedge resection and pulmonary segmentectomy, which was related to the applicability of LMA in short surgical conditions. Compared with the endotracheal intubation group, patients in the LMA group also maintained a satisfactory intraoperative oxygenation, without the risk of hypoxemia, and no significant carbon dioxide accumulation was observed. At the same time, LMA provides a satisfactory operating environment in these particular procedures, without the need for endotracheal or endobronchial intubation. However, the incidence of postoperative throat discomfort and hoarseness was significantly reduced in the LMA group, LMA undoubtedly provides a better subjective feelings and improve postoperative symptoms for these patients.

The results of the sensitivity analysis and meta-analysis were consistent with the conclusion that postoperative airway complications were significantly reduced in the LMA group, and there was no difference in intraoperative airway safety between the two groups. Although, there are differences in ventilation methods and endotracheal tubes, the results of our subgroup analysis were consistent with the pooled results. No ventilation-related adverse events were observed in patients in the LMA group, whether they retained spontaneous ventilation or were on mechanical ventilation. Similarly, compared with the ETT or DLT group, there were no cases of patients in the LMA group requiring endotracheal intubation due to intubation or ventilation failure. We found that LMA can be an alternative to endotracheal intubation for both airway and ventilation management.

Our results are consistent with previous studies, confirming that LMA is associated with a decreased risk of postoperative airway complications [31, 32]. In addition, with the same operation time and anesthesia time, the LMA was confirmed to be safe for minor VATS. The Cochrane’s tool (RoB 2) was used to assess the risk of bias in the included RCTs, and the GRADE tool was used to evaluate the quality of evidence in the included studies. In our study, we conducted a comprehensive and systematic summary of the safety and effectiveness of LMA during VATS. Subgroup analysis and sensitivity analysis were performed to evaluate the robustness of the conclusions, with a large sample size and reliable methodological quality, increasing the generality and representativeness of our conclusions.

With the advancements of comfortable medical treatment and enhanced recovery after surgery, it is equally crucial to pay attention to the perioperative comfortable medical experience of patients from multidimensions and multilevels, as well as to focus on the surgical treatment of the disease itself. A simple and efficient perioperative airway management strategy can not only reduce the incidence of postoperative complications and promote rapid postoperative recovery, but also improve the symptoms and subjective feelings of patients and increase satisfaction, which undoubtedly provides important predictive information for general medical management. Surgical intervention likely plays a decisive role in determining airway management. With the increasing number of VATS procedures performed in outpatient and day surgery centers, the efficacy and safety of LMA has become an important clinical issue.

There is a general consensus that intraoperative airway management during VATS is the primary priority during surgery. DLTs or ETTs combined with bronchial blockers can not only quickly induce artificial pneumothorax, but also provide a capacious space for surgical operation, and ensure continuous ventilation on the nonoperative hemithorax. However, endotracheal intubation may cause mechanical airway injury and lung infection [33, 34]. In addition, postoperative acute airway complications such as airway spasm, vocal cord and laryngeal edema are fairly common [5, 35]. LMA is a kind of supraglottic airway device that can be inserted without laryngoscopy or electronic fiber bronchoscope exposure, and has the advantages of being skillful and having a high success rate [36], offering several superior prospects in airway management over endotracheal intubation. First, LMA causes little damage to throat and pharyngeal tissues, alleviating the endocrine and metabolic changes caused by the tracheal intubation-related stress response, and reducing throat discomfort and hoarseness caused by DLT [37, 38]. Second, patients who underwent LMA were well tolerated, and the preservation of autonomous respiration was more closely related to respiratory physiology, which can promote rapid postoperative recovery [9, 39, 40]. Moreover, the LMA does not require muscle relaxants in some minor VATS, but only a small dose of sedatives and analgesics. Compared with the high doses of sedatives, analgesics and muscle relaxants required for endotracheal intubation and anesthesia maintenance, the dose of intraoperative anesthetics in the LMA group was significantly reduced [29]. After the LMA was completed, an open pneumothorax was induced, and the lung tissue collapsed, which provided a relatively sufficient surgical field for thoracoscopic procedures. The field of thoracoscopic surgery can be increased by reducing the respiratory rate and respiratory movement at the key step of surgery. Of course, these procedures required close cooperation between anesthetists and operators. A lower dose of general anesthetics can reduce the risk of postoperative nausea, vomiting, and gastric retention in patients and prevent aspiration, promoting rapid recovery of gastrointestinal function. Additionally, compression of the respiratory mucosa strongly stimulates sympathetic nerve activity, causing dramatic hemodynamic fluctuations and increasing the burden on the cardiovascular and cerebrovascular systems [41]. LMA prevents the damage of airway mucosa, and is less likely to affect hemodynamics, consequently, it has achieved better results when used for thoracic surgery under anesthesia.

However, compared with endotracheal intubation, LMA also reported the disadvantages and risks. Previous evidence has indicated that patients with LMA, a supraglottic airway device, may have a higher risk of intraoperative gastric insufflation, reflux and aspiration due to its low sealing pressure [42]. Before the pleura is closed, the lungs need to be manually inflated, which may increase the risk of LMA displacement and underventilation. Another concern of adopting LMA is the occurrence of hypoxia or hypercapnia in patients during ipsilateral lung collapse [43], and low tidal volume and high-frequency ventilation are generally used in these processes. Notably, we need to recognize the fact that LMA could increase the respiratory dead space of patients and consequently lead to the accumulation of CO2 in the body, although permissive hypercapnia may prevent lung injury caused by high tidal volume and hyperventilation [44]. Therefore, it is necessary to continuously monitor the PetCO2 and arterial blood partial pressure of carbon dioxide during surgery, and adjust respiratory parameters to promote the discharge of excess CO2.

It should be noted that most of the studies we included were minor VATS, which had short operation time and simple procedures, and often did not have a high demand for lung collapse. Therefore, this conclusion may not be suitable for all thoracic surgery. In major VATS procedures in which the bronchi are often mutilated, the attraction of airway secretions at the end of surgery is especially important when treating such patients. The placement process of an LMA is often complicated, and it is difficult to perform suction under direct visualization. In addition, LMA is also associated with a risk of mucosal injury in a time-dependent manner, which has been verified in animal experiments [18]. However, minor VATS simultaneously avoids the problems of airway secretion suction and the need for a long operation time. For these reasons, LMA may not be recommended for a complicated and time-consuming procedure, while minor VATS procedures such as wedge resection, pleural biopsy, and endoscopic thoracic sympathicotomy may be advantageous over endotracheal intubation.

However, certain limitations of this systematic review and meta-analysis must be highlighted. First, the regions of studies performed are not universally distributed and mainly from China, it may not be applicable for global promotion. Second, there are various VATSs, we cannot perform subgroup analysis according to specific types, but can only roughly classify according to the different type of tubes and ventilation options. Third, positive results were more likely to be published, and the risk of reporting bias should not be underestimated. Finally, the application of LMA in VATS depends significantly on the body habitus, the experience of the operator and assistant, and the depth of anaesthesia. But none of our included studies reported the influence of body habitus and the experience of the operator and assistant on airway management. So, in the future, better RCTs should include such data.

In conclusion, we demonstrated that LMA can effectively reduce postoperative airway complications and promote ERAS post-VATS, our systematic review provided moderate evidence that LMA appears to be an alternative to endotracheal intubation for minor VATS. LMA did not increase the incidence of intraoperative hypoxemia or hypercapnia. In addition, compared with those in the endotracheal intubation group, the LMA group had a lower incidence of postoperative throat discomfort and hoarseness, as well as faster postoperative awake time post-VATS. However, as the quality of evidence from the included studies was moderate and the sample size was small, larger studies with higher quality are needed to confirm our findings.

### Electronic supplementary material

Below is the link to the electronic supplementary material.

Supplementary Material 1

Supplementary Material 2

Supplementary Material 3

Supplementary Material 4

## Data Availability

No datasets were generated or analysed during the current study.

## Abbreviations

VATS: Video-assisted thoracic surgery
LMA: Laryngeal mask airway
DLTs: Double-lumen tubes
ETTs: Endotracheal tubes
SpO2: Pulse blood oxygen saturation
PetCO2: Peak of end tidal CO2 pressure
ICU: Intensive care unit
NOS: Newcastle-ottawa scale
MD: Mean difference
CI: Confidence interval
SMD: Standardized mean difference
RR: Relative risk
RCT: Randomized controlled trial
BB: Bronchial blocker
