# Supplementary material for: Clinical evaluation of laryngeal mask airways in video-assisted thoracic surgery: a meta-analysis of randomized controlled trials
Source: J Cardiothorac Surg. 2024 Jun 24;19:361. doi: 10.1186/s13019-024-02840-6 (PMC11194903; doi:10.1186/s13019-024-02840-6)
Supplement: Supplementary file 1 — Supplementary Material 1 [file 13019_2024_2840_MOESM1_ESM.docx]

Detailed search strategies from PubMed, Embase, Cochrane Library, Medline and Web of Science. The searches will be rerun prior to the final analyses and any further studies identified will be included.

| **Search terms for PubMed** | |
| --- | --- |
| **Search** | Query |
| **#1** | "Laryngeal Mask"[MeSH Terms] |
| **#2** | "Laryngeal Mask"[Title/Abstract] OR "Mask, Laryngeal"[Title/Abstract] OR "Masks, Laryngeal"[Title/Abstract] OR "Laryngeal Mask Airway"[Title/Abstract] OR "Airway, Laryngeal Mask"[Title/Abstract] OR "Airways, Laryngeal Mask"[Title/Abstract] OR " Laryngeal Mask Airways"[Title/Abstract] |
| **#3** | **#1** OR **#2** |
| **#4** | "Thoracic Surgical Procedures"[MeSH Terms] |
| **#5** | "Thoracic Surgical Procedures"[Title/Abstract] OR "Procedures, Thoracic Surgical"[Title/Abstract] OR "Surgical Procedures, Thoracic"[Title/Abstract] OR "Thoracic Surgical Procedure"[Title/Abstract] OR "Procedure, Thoracic Surgical"[Title/Abstract] OR "Surgical Procedure, Thoracic"[Title/Abstract] |
| **#6** | **#4** OR **#5** |
| **#7** | "Thoracic Surgery, Video-Assisted"[MeSH Terms] |
| **#8** | "Thoracic Surgery, Video-Assisted"[Title/Abstract] OR "Surgeries, Video-Assisted Thoracic"[Title/Abstract] OR "Surgery, Video-Assisted Thoracic"[Title/Abstract] OR "Thoracic Surgeries, Video-Assisted"[Title/Abstract] OR "Thoracic Surgery, Video Assisted"[Title/Abstract] OR "Video-Assisted Thoracic Surgeries"[Title/Abstract] OR "Video-Assisted Thoracoscopic Surgery"[Title/Abstract] OR "Surgeries, Video-Assisted Thoracoscopic"[Title/Abstract] OR "Surgery, Video-Assisted Thoracoscopic"[Title/Abstract] OR "Thoracoscopic Surgeries, Video-Assisted"[Title/Abstract] OR "Thoracoscopic Surgery, Video-Assisted"[Title/Abstract] OR "Video Assisted Thoracoscopic Surgery"[Title/Abstract] OR "Video-Assisted Thoracoscopic Surgeries"[Title/Abstract] OR "Video-Assisted Thoracic Surgery"[Title/Abstract] OR "Video Assisted Thoracic Surgery"[Title/Abstract] OR "Surgery, Thoracic, Video-Assisted"[Title/Abstract] OR "VATS"[Title/Abstract] OR "VATSs"[Title/Abstract] |
| **#9** | **#7** OR **#8** |
| **#10** | **#6** OR **#9** |
| **#11** | **#3** AND **#10** |

| **Search terms for EMBASE** | |
| --- | --- |
| **Search** | Query |
| **#1** | ‘Laryngeal Mask’/exp |
| **#2** | ‘Laryngeal Mask’: ab, ti |
| **#3** | ‘Mask, Laryngeal’: ab, ti |
| **#4** | ‘Masks, Laryngeal’: ab, ti |
| **#5** | ‘Laryngeal Mask Airway’: ab, ti |
| **#6** | ‘Airway, Laryngeal Mask’: ab, ti. |
| **#7** | ‘Airways, Laryngeal Mask’: ab, ti |
| **#8** | ‘Laryngeal Mask Airways’: ab, ti. |
| **#9** | **#1** OR **#2** OR **#3** OR **#4** OR **#5** OR **#6** OR **#7** OR **#8** OR **#10** |
| **#10** | ‘Thoracic Surgical Procedures’/exp |
| **#11** | ‘Procedures, Thoracic Surgical’: ab, ti. |
| **#12** | ‘Surgical Procedures, Thoracic’: ab, ti. |
| **#13** | ‘Thoracic Surgical Procedure’: ab, ti. |
| **#14** | ‘Procedure, Thoracic Surgical’: ab, ti. |
| **#15** | ‘Surgical Procedure, Thoracic’: ab, ti. |
| **#16** | **#10** OR **#11** OR **#12** OR **#13** OR **#14** OR **#15** |
| **#17** | ‘Thoracic Surgery, Video-Assisted’ /exp |
| **#18** | ‘Thoracic Surgery, Video-Assisted’: ab, ti. |
| **#19** | ‘Surgeries, Video-Assisted Thoracic’: ab, ti. |
| **#20** | ‘Surgery, Video-Assisted Thoracic’: ab, ti. |
| **#21** | ‘Thoracic Surgeries, Video-Assisted’: ab, ti |
| **#22** | ‘Thoracic Surgery, Video Assisted’: ab, ti |
| **#23** | ‘Video-Assisted Thoracic Surgeries’: ab, ti. |
| **#24** | ‘Video-Assisted Thoracoscopic Surgery’: ab, ti. |
| **#25** | ‘Surgeries, Video-Assisted Thoracoscopic’: ab, ti. |
| **#26** | ‘Surgery, Video-Assisted Thoracoscopic’: ab, ti. |
| **#27** | ‘Thoracoscopic Surgeries, Video-Assisted’: ab, ti. |
| **#28** | ‘Thoracoscopic Surgery, Video-Assisted’: ab, ti. |
| **#29** | ‘Video Assisted Thoracoscopic Surgery’: ab, ti. |
| **#30** | ‘Video-Assisted Thoracoscopic Surgeries’: ab, ti. |
| **#31** | ‘Video-Assisted Thoracic Surgery’: ab, ti. |
| **#32** | ‘Video Assisted Thoracic Surgery’: ab, ti. |
| **#33** | ‘Surgery, Thoracic, Video-Assisted’: ab, ti. |
| **#34** | ‘VATS’: ab, ti. |
| **#35** | ‘VATSs’: ab, ti. |
| **#36** | **#17** OR **#18** OR **#19** OR **#20** OR **#21** OR **#22 OR #23** OR **#24** OR **#25** OR **#26** OR **#27** OR **#28** OR **#29** OR **#30** OR **#31** OR **#32** OR **#33** OR **#34** OR **#35** |
| **#37** | **#16** OR **#36** |
| **#38** | **#9** AND **#37** |

| **Search terms for the Cochrane Library (in Title, Abstract, Keyword)** | |
| --- | --- |
| **Search** | Query |
| **#1** | MeSH descriptor: [Laryngeal Mask] explode all trees |
| **#2** | Laryngeal Mask |
| **#3** | Mask, Laryngeal |
| **#4** | Masks, Laryngeal |
| **#5** | Laryngeal Mask Airway |
| **#6** | Airway, Laryngeal Mask |
| **#7** | Airways, Laryngeal Mask |
| **#8** | Laryngeal Mask Airways |
| **#9** | **#1** OR **#2** OR **#3** OR **#4** OR **#5** OR **#6** OR **#7** OR **#8** |
| **#10** | MeSH descriptor: [Thoracic Surgical Procedures] explode all trees |
| **#11** | Thoracic Surgical Procedures |
| **#12** | Procedures, Thoracic Surgical |
| **#13** | Surgical Procedures, Thoracic |
| **#14** | Thoracic Surgical Procedure |
| **#15** | Procedure, Thoracic Surgical |
| **#16** | Surgical Procedure, Thoracic |
| **#17** | **#10** OR **#11** OR **#12** OR **#13** OR **#14** OR **#15** OR **#16** |
| **#18** | MeSH descriptor: [Thoracic Surgery, Video-Assisted] explode all trees |
| **#19** | Thoracic Surgery, Video-Assisted |
| **#20** | Surgeries, Video-Assisted Thoracic |
| **#21** | Surgery, Video-Assisted Thoracic |
| **#22** | Thoracic Surgeries, Video-Assisted |
| **#23** | Thoracic Surgery, Video Assisted |
| **#24** | Video-Assisted Thoracic Surgeries |
| **#25** | Video-Assisted Thoracoscopic Surgery |
| **#26** | Surgeries, Video-Assisted Thoracoscopic |
| **#27** | Surgery, Video-Assisted Thoracoscopic |
| **#28** | Thoracoscopic Surgeries, Video-Assisted |
| **#29** | Thoracoscopic Surgery, Video-Assisted |
| **#30** | Video Assisted Thoracoscopic Surgery |
| **#31** | Video-Assisted Thoracoscopic Surgeries |
| **#32** | Video-Assisted Thoracic Surgery |
| **#33** | Video Assisted Thoracic Surgery |
| **#34** | Surgery, Thoracic, Video-Assisted |
| **#35** | VATS |
| **#36** | VATSs |
| **#37** | **#18** OR **#19** OR **#20** OR **#21** OR **#22** OR **#23** OR **#24** OR **#25** OR **#26** OR **#27** OR **#28** OR **#29** OR **#30** OR **#31** OR **#32** OR **#33** OR **#34** OR **#35** OR **#36** |
| **#38** | **#17** OR **#37** |
| **#39** | **#9** AND **#38** |
| **Search terms for MEDLINE** | |
| **Search** | Query |
| **#1** | Laryngeal Mask. ab, ti. |
| **#2** | Mask, Laryngeal. ab, ti. |
| **#3** | Masks, Laryngeal. ab, ti. |
| **#4** | Laryngeal Mask Airway. ab, ti. |
| **#5** | Airway, Laryngeal Mas. ab, ti. |
| **#6** | Airways, Laryngeal Mask. ab, ti. |
| **#7** | Laryngeal Mask Airways. ab, ti. |
| **#8** | **#1** OR **#2** OR **#3** OR **#4** OR **#5** OR **#6** OR **#7** |
| **#9** | Thoracic Surgical Procedures. ab, ti. |
| **#10** | Procedures, Thoracic Surgical. ab, ti. |
| **#11** | Surgical Procedures, Thoracic. ab, ti. |
| **#12** | Thoracic Surgical Procedure. ab, ti. |
| **#13** | Procedure, Thoracic Surgical. ab, ti. |
| **#14** | Surgical Procedure, Thoracic. ab, ti. |
| **#15** | **#9** OR **#10** OR **#11** OR **#12** OR **#13** OR **#14** |
| **#16** | Thoracic Surgery, Video-Assisted. ab, ti. |
| **#17** | Surgeries, Video-Assisted Thoracic. ab, ti. |
| **#18** | Surgery, Video-Assisted Thoracic. ab, ti. |
| **#19** | Thoracic Surgeries, Video-Assisted. ab, ti. |
| **#20** | Thoracic Surgery, Video Assisted. ab, ti. |
| **#21** | Video-Assisted Thoracic Surgeries. ab, ti. |
| **#22** | Video-Assisted Thoracoscopic Surgery. ab, ti. |
| **#23** | Surgeries, Video-Assisted Thoracoscopic. ab, ti. |
| **#24** | Surgery, Video-Assisted Thoracoscopic. ab, ti. |
| **#25** | Thoracoscopic Surgeries, Video-Assisted. ab, ti. |
| **#26** | Thoracoscopic Surgery, Video-Assisted. ab, ti. |
| **#27** | Video Assisted Thoracoscopic Surgery. ab, ti. |
| **#28** | Video-Assisted Thoracoscopic Surgeries. ab, ti. |
| **#29** | Video-Assisted Thoracic Surgery. ab, ti. |
| **#30** | Video Assisted Thoracic Surgery. ab, ti. |
| **#31** | Surgery, Thoracic, Video-Assisted. ab, ti. |
| **#32** | VATS. ab, ti. |
| **#33** | VATSs. ab, ti. |
| **#34** | **#16** OR **#17** OR **#18** OR **#20** OR **#21** OR **#22** OR **#23** OR **#24** OR **#25** OR **#26** OR **#27** OR **#28** OR **#29** OR **#30** OR **#31** OR **#32** OR **#33** |
| **#35** | **#15** OR **#34** |
| **#36** | **#8** AND **#35** |

| **Search terms for Web of Science** | |
| --- | --- |
| **Search** | Query |
| **#1** | Laryngeal Mask (Topic) OR Laryngeal Mask (Title) OR Laryngeal Mask (Abstract) OR Mask, Laryngeal (Topic) OR Mask, Laryngeal (Title) OR Mask, Laryngeal (Abstract) OR Masks, Laryngeal (Topic) OR Masks, Laryngeal (Title) OR Masks, Laryngeal (Abstract) OR Laryngeal Mask Airway (Topic) OR Laryngeal Mask Airway (Title) OR Laryngeal Mask Airway (Abstract) OR Airway, Laryngeal Mask (Topic) OR Airway, Laryngeal Mask (Title) OR Airway, Laryngeal Mask (Abstract) OR Airways, Laryngeal Mask (Topic) OR Airways, Laryngeal Mask (Title) OR Airways, Laryngeal Mask (Abstract) OR Laryngeal Mask Airways (Topic) OR Laryngeal Mask Airways (Title) OR Laryngeal Mask Airways (Abstract) |
| **#2** | Thoracic Surgical Procedures (Topic) OR Thoracic Surgical Procedures (Title) OR Thoracic Surgical Procedures (Abstract) OR Procedures, Thoracic Surgical (Topic) OR Procedures, Thoracic Surgical (Title) OR Procedures, Thoracic Surgical (Abstract) OR Surgical Procedures, Thoracic (Topic) OR Surgical Procedures, Thoracic (Title) OR Surgical Procedures, Thoracic (Abstract) OR Thoracic Surgical Procedure (Topic) OR Thoracic Surgical Procedure (Title) OR Thoracic Surgical Procedure (Abstract) OR Procedure, Thoracic Surgical (Topic) OR Procedure, Thoracic Surgical (Title) OR Procedure, Thoracic Surgical (Abstract) OR Surgical Procedure, Thoracic (Topic) OR Surgical Procedure, Thoracic (Title) OR Surgical Procedure, Thoracic (Abstract) |
| **#3** | Thoracic Surgery, Video-Assisted (Topic) OR Thoracic Surgery, Video-Assisted (Title) OR Thoracic Surgery, Video-Assisted (Abstract) OR Surgeries, Video-Assisted Thoracic (Topic) OR Surgeries, Video-Assisted Thoracic (Title) OR Surgeries, Video-Assisted Thoracic (Abstract) OR Surgery, Video-Assisted Thoracic (Topic) OR Surgery, Video-Assisted Thoracic (Title) OR Surgery, Video-Assisted Thoracic (Abstract) OR Thoracic Surgeries, Video-Assisted (Topic) OR Thoracic Surgeries, Video-Assisted (Title) OR Thoracic Surgeries, Video-Assisted (Abstract) OR Thoracic Surgery, Video Assisted (Topic) OR Thoracic Surgery, Video Assisted (Title) OR Thoracic Surgery, Video Assisted (Abstract) OR Video-Assisted Thoracic Surgeries (Topic) OR Video-Assisted Thoracic Surgeries (Title) OR Video-Assisted Thoracic Surgeries (Abstract) OR Video-Assisted Thoracoscopic Surgery (Topic) OR Video-Assisted Thoracoscopic Surgery (Title) OR Video-Assisted Thoracoscopic Surgery (Abstract) OR Surgeries, Video-Assisted Thoracoscopic (Topic) OR Surgeries, Video-Assisted Thoracoscopic (Title) OR Surgeries, Video-Assisted Thoracoscopic (Abstract) OR Surgery, Video-Assisted Thoracoscopic (Topic) OR Surgery, Video-Assisted Thoracoscopic (Title) OR Surgery, Video-Assisted Thoracoscopic (Abstract) OR Thoracoscopic Surgeries, Video-Assisted (Topic) OR Thoracoscopic Surgeries, Video-Assisted (Title) OR Thoracoscopic Surgeries, Video-Assisted (Abstract) OR Thoracoscopic Surgery, Video-Assisted (Topic) OR Thoracoscopic Surgery, Video-Assisted (Title) OR Thoracoscopic Surgery, Video-Assisted (Abstract) OR Video Assisted Thoracoscopic Surgery (Topic) OR Video Assisted Thoracoscopic Surgery (Title) OR Video Assisted Thoracoscopic Surgery (Abstract) OR Video-Assisted Thoracoscopic Surgeries (Topic) OR Video-Assisted Thoracoscopic Surgeries (Title) OR Video-Assisted Thoracoscopic Surgeries (Abstract) OR Video-Assisted Thoracic Surgery (Topic) OR Video-Assisted Thoracic Surgery (Title) OR Video-Assisted Thoracic Surgery (Abstract) OR Video Assisted Thoracic Surgery (Topic) OR Video Assisted Thoracic Surgery (Title) OR Video Assisted Thoracic Surgery (Abstract) OR Surgery, Thoracic, Video-Assisted (Topic) OR Surgery, Thoracic, Video-Assisted (Title) OR Surgery, Thoracic, Video-Assisted (Abstract) OR VATS (Topic) OR VATS (Title) OR VATS (Abstract) OR VATSs (Topic) OR VATSs (Title) OR VATSs (Abstract) |
| **#4** | **#2** OR **#3** |
| **#5** | **#1** AND **#4** |
