# Supplementary material for: Clinical evaluation of laryngeal mask airways in video-assisted thoracic surgery: a meta-analysis of randomized controlled trials
Source: J Cardiothorac Surg. 2024 Jun 24;19:361. doi: 10.1186/s13019-024-02840-6 (PMC11194903; doi:10.1186/s13019-024-02840-6)
Supplement: Supplementary file 2 — Supplementary Material 2 [file 13019_2024_2840_MOESM2_ESM.pdf]

Summary of findings:

LMA compared to DLT/ETT for VATS

Patient or population: VATS
Setting: Inpatient
Intervention: LMA
Comparison: DLT/ETT

Outcomes	Anticipated absolute effects* (95% CI)		Relative effect (95% CI)	№ of participants (studies)	Certainty of the evidence (GRADE)	Comments
	Risk with [对照]	Risk with [干预]				
Postoperative hospital stay	–	SMD **0.47 SD lower** (0.98 lower to 0.03 higher)	–	542 (5 RCTs)	⨁⨁◯◯ Low[a,b]	a. high risk of bias; b. significant heterogeneity
Throat discomfort	349 per 1,000	**98 per 1,000** (59 to 167)	**RR 0.28** (0.17 to 0.48)	642 (6 RCTs)	⨁⨁⨁⨁ High	–
Hoarseness	292 per 1,000	**105 per 1,000** (47 to 236)	**RR 0.36** (0.16 to 0.81)	522 (5 RCTs)	⨁⨁⨁◯ Moderate[b]	b. significant heterogeneity
Postoperative awake time	–	SMD **2.19 SD lower** (3.49 lower to 0.89 lower)	–	544 (5 RCTs)	⨁⨁◯◯ Low[a,b,]	a. high risk of bias; b. significant heterogeneity
Intraoperative minimum SpO2	–	SMD **0 SD** (0.49 lower to 0.49 higher)	–	167 (2 RCTs)	⨁⨁◯◯ Low[a,b,c]	a. high risk of bias; b. significant heterogeneity; c. small sample size
Hypoxemia	25 per 1,000	**25 per 1,000** (7 to 99)	**RR 1.00** (0.26 to 3.89)	315 (2 RCTs)	⨁⨁⨁⨁ High	–
Intraoperative highest PetCO2	–	SMD **0.51 SD higher** (0.12 lower to 1.15 higher)	–	227 (3 RCTs)	⨁⨁⨁◯ Moderate[b]	b. significant heterogeneity
Surgical field satisfaction	979 per 1,000	**989 per 1,000** (960 to 1,000)	**RR 1.01** (0.98 to 1.03)	569 (5 RCTs)	⨁⨁⨁⨁ High	–
Anesthesia time	–	SMD **0.1 SD lower** (0.3 lower to 0.1 higher)	–	590 (6 RCTs)	⨁⨁⨁⨁ High	–
Operation time	–	SMD **0.06 SD higher** (0.13 lower to 0.24 higher)	–	697 (7 RCTs)	⨁⨁⨁⨁ High	–
Blood loss	–	SMD **0.13 SD lower** (0.33 lower to 0.07 higher)	–	384 (3 RCTs)	⨁⨁⨁⨁ High	–

*__The risk in the intervention group__ (and its 95% confidence interval) is based on the assumed risk in the comparison group and the **relative effect** of the intervention (and its 95% CI).
CI: confidence interval; **RR:** risk ratio; **SMD:** standardised mean difference

GRADE Working Group grades of evidence High certainty: we are very confident that the true effect lies close to that of the estimate of the effect. **Moderate certainty:** we are moderately confident in the effect estimate: the true effect is likely to be close to the estimate of the effect, but there is a possibility that it is substantially different. **Low certainty:** our confidence in the effect estimate is limited: the true effect may be substantially different from the estimate of the effect. **Very low certainty:** we have very little confidence in the effect estimate: the true effect is likely to be substantially different from the estimate of effect.

Explanations
a. high risk of bias
b. significant heterogeneity
c. small sample size
